# A minimal mechanically consistent model of smoothly dividing disk-shaped cells

**DOI:** 10.1038/s41540-026-00756-6

**Published:** 2026-06-23

**Authors:** Lukas Hupe, Yoav G. Pollack, Jonas Isensee, Aboutaleb Amiri, Ramin Golestanian, Philip Bittihn

**Affiliations:** 1 Max Planck Institute for Dynamics and Self-Organization, Göttingen, Germany; 2 Institute for the Dynamics of Complex Systems, Göttingen University, Göttingen, Germany; 3 Max Planck Institute for the Physics of Complex Systems, Dresden, Germany; 4 Rudolf Peierls Centre for Theoretical Physics, University of Oxford, Oxford, UK; 5 Present Address: Carl Zeiss, Oberkochen, Germany

**Keywords:** Biophysics, Computational biology and bioinformatics, Mathematics and computing, Physics

## Abstract

Replication through cell division is one of the fundamental processes of life and a major driver of dynamics in systems ranging from bacterial colonies to embryogenesis, tissues and tumors. While regulation also shapes self-organization, many biologically relevant behaviors arise from a limited number of physical ingredients, and particle-based models have become a popular platform to investigate these emergent dynamics. However, incorporating division into such models often produces aberrant mechanical fluctuations that hinder meaningful analysis. Here, we introduce a minimal model ensuring mechanical consistency during cell division. Cells consist of two nodes, overlapping disks which separate during division, forming transient dumbbell shapes. Internal degrees of freedom, cell-cell interactions and equations of motion guarantee force continuity at all times, including during division, both for the dividing cell and its interaction partners, while allowing arbitrary anisotropic mobilities. As a benchmark, we also translate an established model of proliferating spherocylinders with similar dynamics into our theoretical framework. Numerical simulations demonstrate force continuity of the new disk cell model, quantify the improvements, and show agreement in terms of collective behaviors such as alignment and orientational order. We also demonstrate force extraction and a Voronoi-based interpretation in a confluent-tissue context—with a three-dimensional generalization in embryonic-like confinement. A reference implementation of the model in two and three dimensions is freely available as a Julia package based on *InPartS.jl*. Our model provides a framework for analyzing mechanical observables such as velocities and stresses, and can be readily extended with additional biological features.

## Introduction

Multicellular systems ranging from bacterial colonies to tissues, tumors, morphogenesis, and beyond exhibit a wide variety of self-organization phenomena and collective behavior. These systems are driven out of equilibrium by different sources of activity, such as chemical reactions, signaling, metabolism, motility, and proliferation^1,2^. While many of these activities have mechanical consequences, proliferation—the growth and division of cells—is a particularly interesting case: Since it is an indispensable prerequisite for life, it must be relevant for any biological system at a certain stage of its life cycle. It involves, on a certain level of description, the creation and turnover of matter, often violating number and volume conservation, thereby generating internal stresses and large-scale flows. In tissues and developing embryos, such growth-induced stresses and confinement also underlie homeostatic regulation and morphogenesis^3–10^. Proliferation can present a challenge as it usually enters the mathematical description at a fundamental level, violating common assumptions. Besides the direct relevance of mechanical stresses for regulating proliferation^11–16^, many examples of self-organization in multicellular systems arise from mechanical interactions, such as orientational order^17–22^, topological defects^23–27^ and collective motion^27–30^. Given the plethora of interesting phenomena that can already be observed in relatively abstract physical models, a minimal yet consistent mathematical description of the mechanics during growth and division is therefore important to characterize these behaviors. Systems biology traditionally integrates diverse modalities—gene regulation, metabolism, signaling, and various omics data—to understand biological function. Given the growing evidence for the importance of mechanics and physics in shaping cell and tissue behavior, mechanical interactions represent another essential modality that must be incorporated into comprehensive models of multicellular systems. At the multicellular scale, this requires mechanical descriptions that are minimal yet consistent: detailed enough to capture the collective phenomena arising from growth and division, but not so complex as to become intractable when combined with other biological processes. We therefore focus here on the theoretical and numerical underpinnings of such a mechanically consistent model instead of investigating a particular phenomenon, although we simulate some paradigmatic examples of collective behavior.

Depending on the level of detail and the type of questions addressed, multicellular systems can be modeled in different ways in order to study the collective dynamics. Theoretical models often use coarse-grained descriptions such as continuum fields, which allow analytical calculations and approximations of some observables, such as order parameters, or scaling laws, which aid the construction of phase diagrams and studies of phase behavior^31–34^. Examples are continuum models of growing active nematics^19,23^ and scaling theories for tissue growth and regeneration^9,31,35,36^. Vertex models represent the system as a network of vertices connected by edges and implicitly assume confluency, where the dynamics of cell area, perimeter, and junctions are usually determined by an effective free energy, plus possible active contributions^37^. They have, for example, been employed to elucidate the dynamics of epithelial tissues^38^, tumor invasion^39^ and jamming transitions^7^. Another type of abstraction is the division of available space into a lattice, leading to cellular-automaton-type models such as the cellular Potts model, which has been employed to model tumor growth^40^ and morphogenesis^41^. In particle-based models, each particle corresponds to a cell or a part of a cell, and interacts with other particles through explicit forces or potentials. A spectrum of models of different complexity and spirit has been used to investigate multicellular systems. Recent directions include the extension of active-Brownian-particle-like models with attraction and repulsion to model cohesive cell monolayers^42^, with contact inhibition of locomotion leading to emergent structures^43^, entirely athermal models incorporating non-trivial internal cell cycle dynamics^44^ or nearly incompressible cells that are able to recapitulate nematic properties and large-scale orientational order in bacterial colonies^17,20^. These kinds of extensions are easily possible in particle-based models, because they rely less on implicit quantities and effective energies than, e.g., vertex models or continuum models. Additionally, while vertex models are widely used in 2D epithelia, extending them robustly to 3D bulk tissues remains challenging (although significant progress has recently been made in several directions, including incorporating cell migration, cell division and mechanical interactions with the environment^45–47^); an explicit particle-based formulation with post hoc Voronoi mapping provides a natural alternative route in 3D. Independent of any additional mechanisms, however, particle-based models always require a “mechanical backbone”, which defines how different cells interact sterically. This backbone is what we focus on in this study for the case of growth and division, which inevitably have mechanical consequences.

In principle, because of their explicit formulation, incorporating explicit growth and division processes in particle-based models is straightforward (as shown in some of the examples above). However, these processes also come with unique challenges. Most importantly, traditional methods of implementing the process of division often involve instantaneous and abrupt changes in the system, such as the sudden insertion of particles in new locations^4,48,49^, resulting in a discontinuity of forces, or the loss of cell identity. Such artifacts can become problematic in two distinct scenarios: First, when biological processes such as growth or cell death are coupled to mechanical loads—as observed in tumor spheroids^15,16^, developing tissues^11^, and yeast^13^—force discontinuities and stress peaks at division can feed back on cell behavior in unphysical ways. Second, when simulations aim to reproduce experimentally measured mechanical observables such as stress fields^50,51^ or flow patterns^52^, artifacts from division events can corrupt quantitative comparisons. Here, we build a particle-based model of dividing disk-shaped cells with an emphasis on the smoothness of all involved processes. Our goal is to avoid these unphysical mechanical fluctuations as much as possible, providing a framework that can either be employed directly in future studies of stress-dependent couplings or serve as a reference to gauge the impact of discontinuities in other models.

As in many particle-based models of proliferation, we assume that cells always start from the same shape immediately after birth, which is chosen here to be radially symmetric for simplicity (corresponding to an aspect ratio of 1). Binary division inevitably requires some degree of transient anisotropy, which may appear already during the growth phase or, at the latest, just before division. In the first case, the anisotropy manifests as the direction of elongation and thus an anisotropic shape change of the cell towards aspect ratio 2, before it divides into two particles of the original shape. In the second case, all anisotropy is contained in the choice of the division plane, i.e. the axis along which the two daughter cells are placed. However, in this second case, new particles must necessarily be inserted in new places, causing force discontinuities which we are trying to avoid. Hence, we investigate the first case of gradually introducing cell shape anisotropy.

First, we introduce a common set of coordinates and equations of motion which can be used to represent various physical interaction rules for objects with nematic symmetry. It is based on two nodes at opposite poles of the cell, around which specific force laws can be built. Using this formulation and an appropriate force decomposition, we naturally ensure consistent motion of the entire cell as a rigid body in response to external forces and torques, while having the freedom to define arbitrary internal dynamics of the separation between nodes. We then define two sets of interaction forces between the two nodes of a cell and between nodes of different cells: one is our new model of disk cells, which treats individual nodes as disk-shaped elastic objects, and, as a consequence, the entire cell as a dumbbell during elongation. With a few custom modifications, it is possible to achieve a high degree of force continuity in this case. As a comparison, the other set of interaction forces represents cells as spherocylinders—a common choice for rod-shaped bacteria in the literature—which also elongate incrementally and simplify to disks for an aspect ratio 1.

In the second part of the study, we examine and compare both models: We start on the microscopic scale by demonstrating the presence or absence of force continuity and characterize force fluctuations in small growing colonies. We then extend the comparison to collective properties that manifest from these microscopic interactions, using cell orientation and nematic order as a prominent example. The comparison is followed by an application of the disk cell model that demonstrates how to bridge the gap to dense tissue models using Voronoi tessellations in both two and three dimensions. Finally, we conclude by discussing how the unique features of our model will facilitate certain applications and by pointing out possible extensions.

## Results

### Disk cell model

**Fig. 1:**
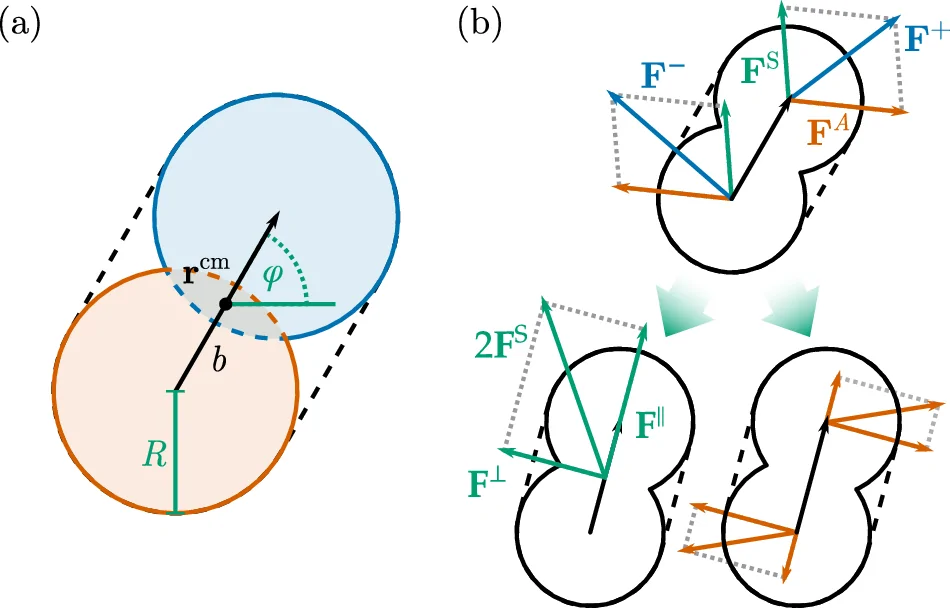
Degrees of freedom and force decomposition for a single cell. **a** Illustration of a single cell, with backbone of length *b*, orientation *φ*, center-of-mass position ${\bf{r}}^{\rm{cm}}$ and radius *R*. Disks are colored and the spherocylinder outline is indicated by dashed lines. **b** Illustration of force decomposition. The symmetric (green, upper panel) and antisymmetric (orange, upper panel) components of the node forces (blue, upper panel) are decomposed into the center-of-mass forces (green, lower panel) and the rotational and internal forces (orange, lower panel).

As the simplest description of an anisotropic particle, we model an individual cell *i* as two nodes connected by a spring of length *b*_*i*_ and orientation *φ*_*i*_, with a center-of-mass position ${{\bf{r}}}_{i}^{{\rm{cm}}}$. Figure 1a shows these degrees of freedom together with the two sets of shapes—disks (colored) and spherocylinders (dashed)—that we will later introduce through appropriate interaction functions.

#### Growth and division

To model the cellular life cycle, every cell has an internal clock *g*_*i*_ ∈ [0, 1) which grows linearly in time with a growth rate ${\mathop{g}\limits^{.}}_{i}={\gamma }_{i}$. Growth is modeled by changing the rest length *b*^eq^ of the internal spring as a function of the internal clock, with

1 $${b}^{{\rm{eq}}}({g}_{i},R)=2R\cdot {g}_{i}\,.$$

At *g*_*i*_ = 0, both nodes overlap completely, while at *g*_*i*_ = 1 they tend to move completely apart. In biological systems, growth depends on a number of external factors such as the availability of nutrients and mechanical stress. The unconditional growth modeled here, therefore, corresponds to a regime where growth is only limited by the internally saturated reproduction rate of cells. For different applications, extensions are easily realizable by replacing *γ* with a stress-dependent growth function^53^, since the disk cell model already provides convenient access to a measure for local stress, as we will point out below in section “Disk cell model: interactions”.

Having reached *g*_*i*_ = 1, cells can now be divided into two equally sized daughter cells, each initialized with internal clocks set to zero, in the positions of the two nodes of the parent cell. To desynchronize growth cycles, we draw the growth rates of these newly born cells independently from a growth rate distribution *P*(*γ*). In the following, we will choose growth rates from a uniform distribution on the interval [0.75*γ*_0_, 1.25*γ*_0_] with a constant *γ*_0_. Cell orientation and all other static parameters are inherited from the parent.

An overview of all static and dynamic model parameters, as well as some of the functions and symbols used to describe the model, can be found in Table 1.

**Table 1:** Overview of model parameters and symbols

Dynamic variables	
${{\bf{r}}}_{i}^{\pm }$	Node positions
${{\bf{r}}}_{i}^{{\rm{cm}}}$	Center-of-mass position
*b*_*i*_	Backbone length
*φ*_*i*_	Cell orientation
*g*_*i*_	Growth phase

Static parameters	
$l^{\rm{max}}$	Division length (fixed to 4*R* for disk cells)
*γ*_*i*_	Growth rate, ${\gamma }_{i}=\frac{{\rm{d}}{g}_{i}}{{\rm{d}}t}$
*R*	Node radius/cell extent from backbone
*Y*	Young’s modulus
*η*	Viscosity

Forces	
${{\bf{F}}}_{i}^{\pm }$	Node forces
${F}_{i}^{\parallel }$	Translational force (longitudinal)
${F}_{i}^{\perp }$	Translational force (transverse)
${F}_{i}^{{\rm{int}}}$	Internal force
*T*_*i*_	Torque

Functions	
*b*^eq^(*g*_*i*_)	Backbone rest length
*μ*^∥^(*b*_*i*_)	Translational mobility (longitudinal)
*μ*^⊥^(*b*_*i*_)	Translational mobility (transverse)
*μ*^rot^(*b*_*i*_)	Rotational mobility
*μ*^int^(*b*_*i*_)	Internal mobility
*P*(*γ*)	Growth rate distribution

A subscript index *i* indicates that the quantity is specific to an individual cell. In node-specific quantities, the superscript + or − indicates the values for the positive and negative node, respectively. The symbols ∥ and ⊥ denote vector components longitudinal or transverse to the cell.

#### Equations of motion

We model the dynamics of the cells with overdamped equations of motion, i.e.

2 $$\frac{{\rm{d}}}{{\rm{d}}t}{\bf{x}}=\underline{{\boldsymbol{\mu }}}\cdot \mathop{\sum }\limits_{\alpha }{{\bf{F}}}_{\alpha }\,,$$

where *μ* is the mobility tensor.

For a single cell with the four degrees of freedom ${{\bf{r}}}_{i}^{{\rm{cm}}}$, *b*_*i*_ and *φ*_*i*_, we can write these equations as

3 $$\frac{{\rm{d}}}{{\rm{d}}t}\,{{\bf{r}}}_{i}^{{\rm{cm}}}={\underline{{\boldsymbol{\mu }}}}^{{\rm{cm}}}({b}_{i},{\varphi }_{i})\cdot {{\bf{F}}}_{i}^{{\rm{cm}}}$$

4 $$\frac{{\rm{d}}}{{\rm{d}}t}\,{b}_{i}={\mu }^{{\rm{int}}}({b}_{i})\cdot {F}_{i}^{{\rm{int}}}$$

5 $$\frac{{\rm{d}}}{{\rm{d}}t}\,{\varphi }_{i}={\mu }^{{\rm{rot}}}({b}_{i})\cdot {T}_{i}.$$

where ${{\bf{F}}}_{i}^{{\rm{cm}}}$ is the force acting on its center of mass, ${F}_{i}^{{\rm{int}}}$ is the force acting on the internal spring, and *T*_*i*_ is the torque with respect to the center of mass. We can simplify Eq. (3) by decomposing the right-hand side into its components parallel and perpendicular to the backbone. This effectively diagonalizes *μ*_cm_, eliminates the angle dependency, and yields

6 $$\frac{{\rm{d}}}{{\rm{d}}t}\,{{\bf{r}}}_{i}^{{\rm{cm}}}={\mu }^{\parallel }({b}_{i})\,{F}_{i}^{\parallel }\cdot {\widehat{{\bf{e}}}}_{i}+{\mu }^{\perp }({b}_{i})\,{F}_{i}^{\perp }\cdot (\widehat{{\bf{z}}}\times {\widehat{{\bf{e}}}}_{i})$$

where ${\widehat{{\bf{e}}}}_{i}=(\cos ({\varphi }_{i}),\sin ({\varphi }_{i})){\rm{T}}$ and the two-dimensional “cross product” is defined as $\widehat{{\bf{z}}}\times {({x}_{1},{x}_{2})}^{{\rm{T}}}={(-{x}_{2},{x}_{1})}^{{\rm{T}}}$.

Our implementation of the model allows for different choices of mobilities, enabling us to model different sources of friction in different applications. Here, we use a numerical approximation for the mobilities of a dumbbell in a viscous fluid, derived by Lüders et al.^54^, to set the external mobilities *μ*^∥^, *μ*^⊥^, and *μ*^rot^ as

7 $${\mu }^{\parallel }(a)=\frac{1}{2\pi \,\eta \,(2R\,a)}\,(\log (a)+{c}_{1}+\,{c}_{2}\,{a}^{-1}+{c}_{3}\,{a}^{-2})$$

8 $${\mu }^{\perp }(a)=\frac{1}{4\pi \,\eta \,(2R\,a)}\,(\log (a)+{c}_{4}+\,{c}_{5}\,{a}^{-1}+{c}_{6}\,{a}^{-2})$$

9 $${\mu }^{{\rm{rot}}}(a)=\frac{3}{\pi \,\eta \,{(2Ra)}^{3}}\,(\log (a)+{c}_{7}\,+\,{c}_{8}\,{a}^{-1}+\,{c}_{9}\,{a}^{-2})$$

where the viscosity *η* controls the magnitude of mobilities, *a* = *b*_*i*_/2*R* + 1 is the aspect ratio of the cell and the coefficients *c*_*i*_ are listed in Table 2. We note, however, that the concrete choice of mobility has little impact on the emergent dynamics in the settings studied in this work, as long as the basic properties like the ensemble average friction are approximately preserved and the rotational mobilities do not take on extreme values. For situations where friction with a two-dimensional substrate dominates, a simpler common choice is

10 $${\mu }^{{\rm{trans}}}(a)=1/(\xi lw)$$

11 $${\mu }^{{\rm{rot}}}(a)=12{\mu }^{\parallel }/({w}^{2}+{l}^{2}),$$

where *ξ* is the friction coefficient (per unit area) and the contact area is approximated through the length *l* = *b*_*i*_ + 2*R* and the width *w* = 2*R* of the enclosing rectangle. In this case, the translational mobility *μ*^trans^ = *μ*^∥^ = *μ*^⊥^ is isotropic. The anisotropic mobilities in Eqs. (7–9) become more relevant for larger division aspect ratios and in three-dimensional simulations, where the surrounding fluid is a more likely source of friction.

**Table 2:** Values of the coefficients used for the mobility laws Eq. (7–9)

Mobility coefficients		
	Dumbbell	Rod
*c*_1_	−0.0552	−0.1404
*c*_2_	0.8477	1.034
*c*_3_	−0.1254	−0.228
*c*_4_	1.025	0.8369
*c*_5_	0.1317	0.5551
*c*_6_	0.178	−0.06066
*c*_7_	−0.3429	−0.3512
*c*_8_	0.7749	0.7804
*c*_9_	−0.09898	−0.09801

To choose a value for the internal mobility *μ*^int^ that is consistent with the external mobilities, we first need to consider how external forces can act on the internal degree of freedom.

#### Force decomposition

An external force acting on a cell can—depending on its impact point and angle—influence all four degrees of freedom (position, orientation, backbone length) simultaneously. For the disk model, all steric forces are modeled as central forces acting on the two nodes located at the positions

12 $${{\bf{r}}}_{i}^{\pm }={{{\bf{r}}}^{{\rm{cm}}}}_{i}\pm \frac{{b}_{i}\,{\widehat{{\bf{e}}}}_{i}}{2}.$$

We call ${{\bf{r}}}_{i}^{\pm }$ the positive (P or +) and negative (N or −) nodes of the cell. Later, we will define all interactions in terms of the force ${{\bf{F}}}_{ij}^{\alpha \beta }$ exerted on the node *α* ∈ { +, − } of a cell *i* by the node *β* of another cell *j*, which we assume to be a function of the inter-node distance vectors. We define the corresponding distance vector from node *β* of cell *j* to node *α* of cell *i* as ${{\bf{d}}}_{ij}^{\alpha \beta }={{\bf{r}}}_{i}^{\alpha }-{{\bf{r}}}_{j}^{\beta }$. This choice means that the backbone vector connecting two nodes of the *same* cell can be expressed as ${b}_{i}{\widehat{{\bf{e}}}}_{i}={{\bf{d}}}_{ii}^{+-}$, and that repulsive forces ${{\bf{F}}}_{ij}^{\alpha \beta }$ are always parallel to their respective distance vectors ${{\bf{d}}}_{ij}^{\alpha \beta }$. However, as the equations of motion require forces that act on the individual degrees of freedom of a cell, at some point, the total force ${{\bf{F}}}_{i}^{\alpha }={\sum }_{\beta ,j}{{\bf{F}}}_{ij}^{\alpha \beta }$ exerted on the node *α* of cell *i* by all other nodes has to be transformed back into these cell coordinates. For this, the first step is to decompose the forces into a symmetric and an antisymmetric component (compare Fig. 1b).

13 $${{\bf{F}}}_{i}^{{\rm{S}}}=\frac{1}{2}({{\bf{F}}}_{i}^{+}+{{\bf{F}}}_{i}^{-})$$

14 $${{\bf{F}}}_{i}^{{\rm{A}}}=\frac{1}{2}({{\bf{F}}}_{i}^{+}-{{\bf{F}}}_{i}^{-}).$$

The force experienced by the center of mass must be equal to the sum of the forces experienced by the nodes, i.e. $2{{\bf{F}}}_{i}^{{\rm{S}}}$. Using the scalar components ${F}_{i}^{\parallel }$ and ${F}_{i}^{\perp }$ of the center-of-mass force (compare Eq. (6)), we can write

15 $${F}_{i}^{\parallel }={\widehat{{\bf{e}}}}_{i}\cdot 2\,{{\bf{F}}}_{i}^{{\rm{S}}}$$

16 $${F}_{i}^{\perp }=(\widehat{{\bf{z}}}\times {\widehat{{\bf{e}}}}_{i})\cdot 2\,{{\bf{F}}}_{i}^{{\rm{S}}}$$

with the cross product $\widehat{{\bf{z}}}\times {\widehat{{\bf{e}}}}_{i}$ as defined in Eq. (6).

An analogous decomposition can be applied to the antisymmetric component: The parallel antisymmetric force will affect the cell length, while the orthogonal antisymmetric force will change the cell orientation. This can be expressed as a scalar force and a torque

17 $${F}_{i}^{{\rm{int}}}={\widehat{{\bf{e}}}}_{i}\cdot 2\,{{\bf{F}}}_{i}^{{\rm{A}}}$$

18 $${T}_{i}=\widehat{{\bf{z}}}\cdot ({b}_{i}{\widehat{{\bf{e}}}}_{i}\times {{\bf{F}}}_{i}^{{\rm{A}}}).$$

Note that the factor of two in Eq. (17) is a matter of convention and can be compensated for when choosing the internal mobility.

We can now examine the effect of the value of the internal mobility *μ*^int^ on the dynamics of the node positions **r**^±^ by considering a simple example: a force ${{\bf{F}}}_{i}^{-}=f\,{\widehat{{\bf{e}}}}_{i}$ is applied to the negative node ${{\bf{r}}}_{i}^{-}$ of a cell at rest (*b*_*i*_ = *b*^eq^(*g*_*i*_)). In the absence of explicit coupling between the two nodes (i.e., without the internal spring), the positive node remains force-free. Using the force decomposition as outlined above, we find ${F}_{i}^{\parallel }=f$ and *F*^int^ = − *f*. We can thus write the velocity of the positive node as

19 $$\begin{array}{l}{\dot{{\bf{r}}}}_{i}^{+}={\dot{{\bf{r}}}}_{i}^{\mathrm{cm}}+\frac{\dot{{b}_{i}}\,{{\bf{e}}}_{i}}{2}\\ =\frac{2{\mu }^{\parallel }-{\mu }^{\mathrm{int}}}{2}\,f\,{{\bf{e}}}_{i}\,.\end{array}$$

From this result, we see that for *μ*^int^ ≠ 2*μ*^∥^, there is an implicit coupling between the nodes that will cause the positive node to move without any force acting on it. In fact, if *μ*^int^ > 2*μ*^∥^, it will move in the opposite direction to the force applied to the cell. Thus, we only consider *μ*^int^ ≤ 2*μ*^∥^ physically meaningful and choose *μ*^int^ = 2*μ*^∥^ as the canonical value.

#### Interactions

In real life, the interactions between cells in a colony or tissue can be fairly complex and include diverse effects such as adhesion and chemical signaling. Although these could in principle be implemented as part of our model, here, we limit ourselves to mechanical repulsion caused by volume exclusion, which we model using pairwise forces between the nodes: two overlapping nodes repel each other with a force derived from Hertzian contact theory for homogeneous elastic spheres, which scales with the node overlap to the power of $\frac{3}{2}$ ^55,56^. Thus, the force exerted on node *α* of a cell *i* by node *β* of another cell *j* is

20 $${{\bf{F}}}_{ij}^{\alpha \beta }={m}_{ij}\frac{Y}{2}\sqrt{\frac{R}{2}}{(2R-\parallel {{\bf{d}}}_{ij}^{\alpha \beta }\parallel )}^{3/2}\frac{{{\bf{d}}}_{ij}^{\alpha \beta }}{\parallel {{\bf{d}}}_{ij}^{\alpha \beta }\parallel }\,{\rm{for}}\,i\ne j,\,{\rm{and}}\,\parallel {{\bf{d}}}_{ij}^{\alpha \beta }\parallel \le 2R$$

where *m*_*ij*_ is a softness factor that compensates for instantaneous node doubling on division (see further below).

The additional factors arise from the transformation of the normal contact problem of two elastic spheres to that of a single hard indenter in an elastic half-space (see ref. ^56^, Section 2.5.3), and are here given for the case of equal node radii and Young’s moduli. The numerical implementation of our model uses the full expressions without this simplification.

To keep steric forces between nodes continuous on division, we use the same Hertzian force law for the internal spring connecting the two nodes

21 $${{\bf{F}}}_{ii}^{\alpha \beta }=\frac{Y}{2}\sqrt{\frac{R}{2}}\cdot {\rm{sgn}}(\Delta {b}_{i})\cdot | \Delta {b}_{i}{| }^{3/2}\,\frac{{{\bf{d}}}_{ii}^{\alpha \beta }}{\parallel {{\bf{d}}}_{ii}^{\alpha \beta }\parallel }\,{\rm{for}}\,\alpha \, \ne \, \beta$$

with Δ*b*_*i*_ = *b*^eq^(*g*_*i*_, *R*) − *b*_*i*_. Note that ${{\bf{d}}}_{ii}^{+-}$ is simply the backbone vector ${b}_{i}{\widehat{{\bf{e}}}}_{i}$ according to our index convention (see beginning of section “Force decomposition”) and therefore $\parallel {{\bf{d}}}_{ii}^{-+}\parallel =\parallel {{\bf{d}}}_{ii}^{+-}\parallel ={b}_{i}$ and ${{\bf{d}}}_{ii}^{+-}/\parallel {{\bf{d}}}_{ii}^{+-}\parallel =-{{\bf{d}}}_{ii}^{-+}/\parallel {{\bf{d}}}_{ii}^{-+}\parallel ={\widehat{{\bf{e}}}}_{i}$ in Eq. (21). Therefore, the functional form of the internal spring and the inter-cell force law in Eqs. (21 and 20) are exactly matched at cell division, when *b*^eq^(*g*_*i*_ = 1, *R*) = 2*R*. This leads to continuous forces when two nodes switch from belonging to the same mother cell, interacting according to Eq. (21), to becoming two distinct daughter cells, each with its own nodes interacting according to Eq. (20), independently of the cell’s compression state. Note that this is not true for the case *b*_*i*_ > *b*^eq^ (i.e., the backbone is expanded instead of relaxed or compressed), since Eq. (20) does not have an attractive regime. However, this case rarely occurs and, if so, could be considered a physical rupture event for which the force jump is actually correct.

As an aside, note that the restoring force ${{\bf{F}}}_{ii}^{\alpha \beta }$ and deviation of the backbone length Δ*b*_*i*_ from its rest length provide convenient access to measures for local longitudinal stress and strain, respectively. Although we will use constant growth rates ${\mathop{g}\limits^{.}}_{i}={\gamma}_{i}$ as defined in section “Growth and division” for our model demonstration below, it would therefore be straightforward to make the growth rate (or other processes like cell removal) dependent on local mechanical cues^53,57^.

The replacement of parent nodes with new child cells introduces another potential source of discontinuity: while child cells take up the exact space occupied by the mother, all forces due to overlap with nodes of neighboring cells will double on division as the single parent node is replaced by the two nodes of the corresponding child cell. Similarly, the forces between the two nodes of the same cell would quadruple when they are replaced by two nodes each in identical positions upon division. To prevent these discontinuities, we include the softness factor *m*_*ij*_ in the force law in Eq. (20) to continuously connect the two situations before and after division, and interpolate between them:

22 $${m}_{ij}=\frac{({g}_{i}+1)\,({g}_{j}+1)}{4}.$$

Each cell *i* effectively contributes a factor (*g*_*i*_ + 1)/2 to this expression, leading to half-strength forces for *g*_*i*_ = 0 directly after division. For cells interacting with the two nodes of a child cell in identical positions, these then add up to the full-strength interaction force with the corresponding node of the mother *k* that was present just before division (when *g*_*k*_ = 1). In combination, for daughter cells *i* and *j* of the same mother cell, *m*_*ij*_ exactly compensates the increase in the number of interactions from 1 internal one to 4 external ones. In between birth and division, *m*_*ij*_ continuously interpolates between the two extremes, increasing the forces from half to full strength.

### Rod cell model

For comparison with a traditional model from the literature featuring incremental elongation, we now specify an alternative set of force laws and slightly modified equations of motion, using the same framework of two nodes (which we will call pseudonodes in the context of this model) and the corresponding force decomposition as defined above. The model (without the translation to our framework) has been commonly used in the literature for both two- and three-dimensional simulations of bacilliform bacteria^18,20,58–61^ and represents cells as discorectangles (i.e., the two-dimensional equivalent of spherocylinders). Here, the cell “surface” is defined as the locus of points with a distance of *R* to the cell backbone, as indicated by the dashed outline in Fig. 1a. Since this model also exhibits gradual elongation with subsequent division, and its parameter space includes the case of cells growing from aspect ratio 1 to 2 before dividing, it is comparable with our disk cell model in many aspects.

Unlike disk cells, this model allows any division length $l^{\rm{max}} \ge 4R$, although we are primarily interested in the lower limit in analogy to the disk cells. The general time evolution of the rest length is

23 $${b}^{\mathrm{eq}}({g}_{i},{l}^{{\mathrm{max}}},R)=\frac{{l}^{{\mathrm{max}}}}{2}\cdot ({g}_{i}+1)-2R\,.$$

In contrast to our disk cell model, the backbone length *b*_*i*_ is commonly not considered a dynamic degree of freedom. In line with this, we will consider the limit of an infinitely stiff internal spring, so that the backbone length always follows *b*^eq^. Note that this requires the introduction of a self-consistent virtual force in the analysis (not the simulation itself), which can be obtained directly from the equations of motion and preserves force balance during the prescribed elongation.

At cell division, the parent cell is replaced by two new cells placed and sized to fill its outline at the time of division. Their lengths add up to that of the removed parent, orientations are geometrically enforced to be identical, and the new center positions must lie at one-fourth and three-fourths of the parent cell’s total length. When a compressed cell divides, its daughter cells are therefore also initialized with appropriate compression. In the case of rod cells with the minimum possible division length of ${l}^{{\mathrm{max}}}=4R$, newly created cells are circular, and their center position coincides with the pseudonodes of the parent as in the dumbbell model.

**Fig. 2:**
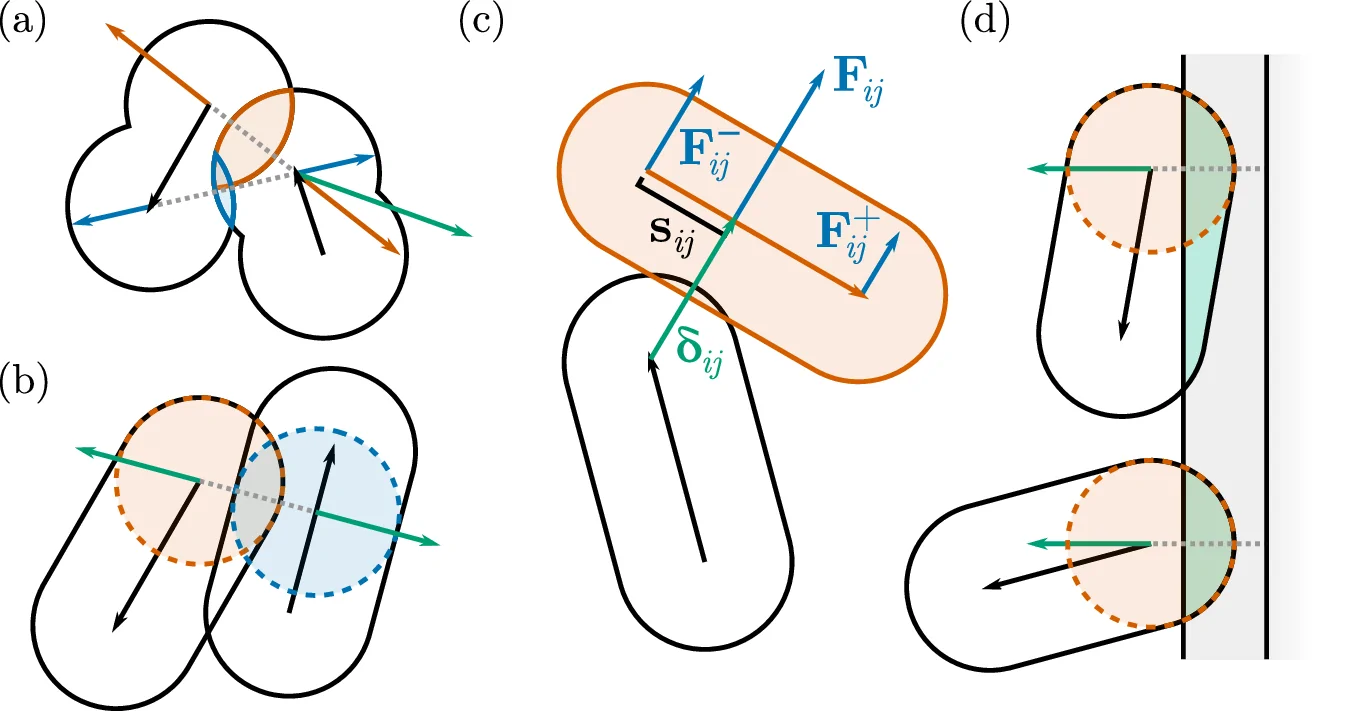
Interaction forces for the two different models. **a** Two interacting disk cells with highlighted node overlaps and forces. **b** Two interacting rod cells with highlighted virtual Hertzian disks and the shortest distance *δ*_*ij*_ (gray dotted line). **c** Illustration of the redistribution of a force **F**_*ij*_ onto the virtual node forces of cell *i* (red). **d** Two short rod cells interacting with a much longer cell, showing that pivoting the cell around one of its nodes can cause the overlap area (shaded in green) to grow dramatically while keeping the forces constant.

Forces depend on the shortest distance vector *δ*_*ij*_ between backbones, with

24 $${{\bf{F}}}_{ij}=\frac{Y}{2}\sqrt{\frac{R}{2}}{(2R-\parallel {\delta }_{ij}\parallel )}^{3/2}\frac{{\delta }_{ij}}{| {\delta }_{ij}| }$$

for ∣*δ*_*ij*_∣ < 2*R* and zero otherwise. This force is distributed onto the pseudonode components according to the relative position 0 ≤ *s*_*ij*_ ≤ 1 of its attack point on the cell backbone (Fig. 2c), using ${{\bf{F}}}_{ij}^{+}={s}_{ij}\,{{\bf{F}}}_{ij}$ and ${{\bf{F}}}_{ij}^{-}=(1-{s}_{ij})\,{{\bf{F}}}_{ij}$. Within the existing framework, this procedure preserves center-of-mass forces and torques.

This single-contact-point scheme is similar in spirit to the Hertzian dumbbell forces, but it has known limitations: the overlap area can change without affecting forces (Fig. 2d), and near-parallel configurations become sensitive to torque fluctuations since the closest point becomes ill-defined. These issues contribute to the force discontinuities of this model analyzed in the section “Force continuity” below.

As with the dumbbells, we use mobilities following Eqs. (7–9), with altered coefficients as given in Table 2 and reported in ref. ^54^ to account for the spherocylindrical shape. These mobilities are in general slightly higher than those for dumbbells, but converge with them for *a* = 1. Likewise, it is also possible to use the simpler contact area-based mobilities according to Eqs. (10) and (11) when appropriate.

### Model comparison and applications

To test whether the disk cell model indeed achieves the desired properties, and how it compares to the alternative rod cell, we carry out simulations which demonstrate the consequences of these modeling choices. In the section “Force continuity”, we will examine the microscopic mechanical interactions and their implications on a population level. Subsequently, in section “Collective dynamics, orientational order, angle statistics”, we turn to orientational dynamics as an example of emergent behavior, and then explore the application of the model to tissue mechanics in section “Connection to tissue modeling in two and three dimensions”.

#### Force continuity

**Fig. 3:**
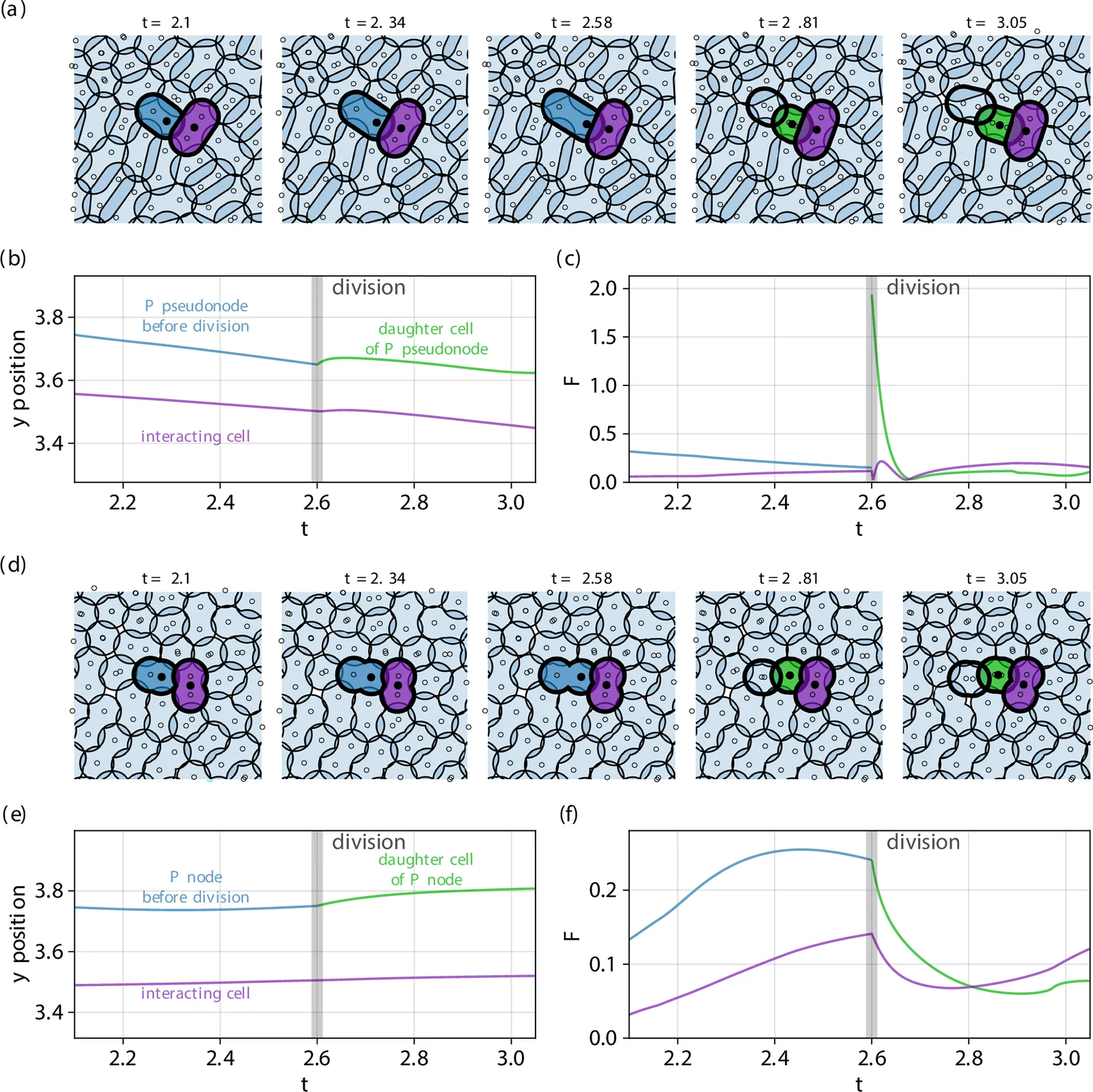
Continuity of positions and forces. **a** Snapshots of a single growing rod cell (blue) immersed in a colony of passive cells. Simulations done using a domain size of 7 × 7 with bi-periodic boundary conditions and parameters *R* = 0.5, *η* = 0.05, *Y* = 50 and $l^{\rm{max}} =2$ with all growth rates set to 0 except one growing particle as described in the text. Tracked objects (marked with filled circles ●): P pseudonode of the growing cell, one cell (purple) interacting with it, and a daughter cell (green) resulting from the P pseudonode. All other pseudonodes are marked with empty circles ○. **b** Positions of tracked entities in the simulation in (**a**). **c** Forces on tracked entities in the simulation in (**a**). **d** Snapshots of a single growing disk cell (blue) immersed in a colony of passive cells. Parameters and tracked objects analogous to (**a**). Daughter cell (green) arises from the tracked P node of mother cell (blue). **e** Positions of tracked entities in the simulation in (**d**). **f** Forces on tracked entities in the simulation in (**d**).

To be able to track inter-particle forces on the microscopic level, we prepare a small simulation in a square domain with a side length of seven node diameters and bi-periodic boundary conditions. We place 49 non-growing particles (*γ*_*i*_ = 0) with random *g*_*i*_ ∈ [0, 1) and random *φ*_*i*_ in the simulation domain, except a single selected particle *k* near the center for which *g*_*k*_ is set to 0.4. After evolving the system for 2.0 time units to relax initial mechanical stresses, the particles are in a configuration with significant overlaps, as visible in the snapshots in Fig. 3. We then set the growth rate of the chosen particle to *γ*_*k*_ = 1 and observe it until after its division at *t* = 2.6 (blue particle with green descendant in Fig. 3a, d).

As a measure for the smoothness of the dynamics, Fig. 3b shows the *y* positions of the P pseudonode of the growing rod cell (blue), of the daughter cell emerging from it (green) and of one interacting cell in the vicinity (purple). While the positions have to be continuous by definition (considering the division protocol and their emergence from the integration of ODEs), a distinct kink at division is visible, indicating an abruptly changing velocity for the daughter cell. Figure 3c indicates a large force jump as the reason for this kink: the daughter cell is placed at the position of the mother cell’s P pseudonode, resulting in an instantaneous loss of the rigid backbone’s support, which compensated for the compressive forces from the surrounding cells. Another significant jump can be seen in the force on the interacting cell due to the instantaneous replacement of the mother cell by differently shaped objects. Both of these forces converge back to levels typical for the overall configuration after a short time of mechanical relaxation.

In contrast, the corresponding plots for the disk cell simulation in Fig. 3d show smooth node trajectories (Fig. 3e) and continuous forces (panel f) without jumps. The continuity between the force on the P node and that on the daughter cell is a result of both our choice of internal spring in Eq. (21), which mimics the external repulsion after division, and the softness factor according to Eq. (22), which avoids instantaneous quadrupling of forces when nodes are replaced by entire cells. This softness factor, together with the fact that the mother cell is replaced by objects which maintain the exact shape from before division, also guarantees force continuity for the interacting cell. Note that a mechanical relaxation process exploring the newly added degrees of freedom is also visible for disk cells, after the two compartments are untethered during division. However, in contrast to rod cells, the forces remain continuous and within the same order of magnitude (compare *y* axis scales in panels c and f).

So far, we have seen that our model definition is able to avoid large jumps for individual interaction forces when an isolated cell divides, while other dynamics in the colony are limited to passive rearrangements. Next, we wanted to explore the consequences of this improvement for an entire growing colony, where fluctuations are expected in any event due to a continuous stream of rearrangements, division and removal events. This could be important for extracting physically meaningful stresses and instantaneous velocities from a simulation.

**Fig. 4:**
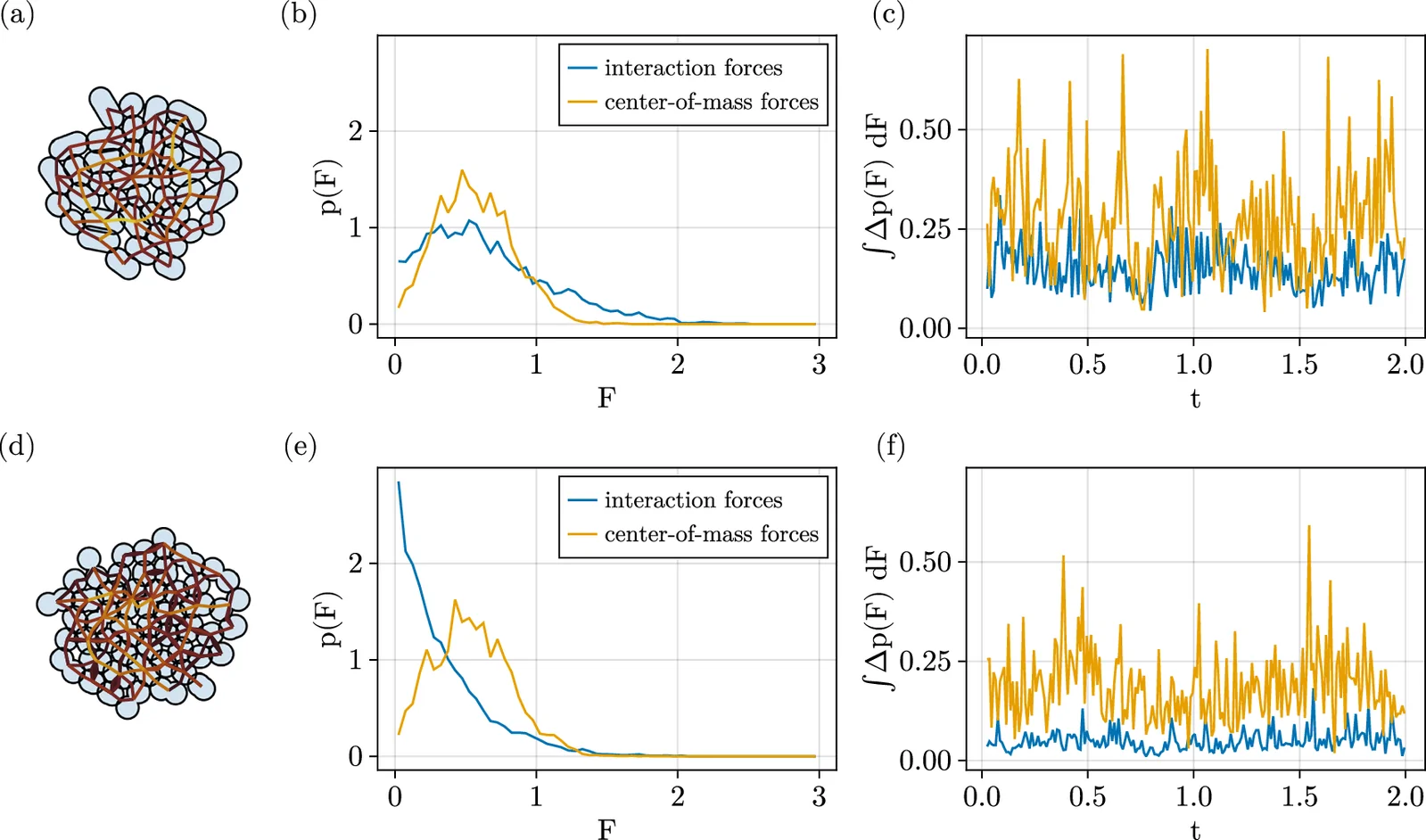
Statistics of interaction forces and center-of-mass forces. **a** Snapshots of a small circular colony of growing rod cells. Simulations done using a circular domain of diameter 8 with absorbing boundary conditions and parameters *R* = 0.5, *η* = 0.05, *Y* = 80 and $l^{\rm{max}}=2$ with growth rates chosen uniformly between 0.75 and 1.25. Lines from cell centers to boundaries indicate intercellular interactions, where brighter colors represent stronger forces. **b** Distribution of individual interaction forces and total center-of-mass forces on each cell in the simulation depicted in (**a**) averaged over 2 generations. **c** Integrated squared difference between consecutive force distributions 0.01 generations apart. **d**–**f** Same as in (**a**–**c**) but for disk cells (with identical simulation parameters).

To investigate this, we set up a small simulation with circular absorbing boundary conditions, that is, cells beyond a certain distance from the center are removed, while all cells continually grow with individual random growth rates *γ*_*i*_ drawn from a uniform distribution on the interval [0.75, 1.25]. Starting with a few initial cells, the domain quickly fills, and the simulations enter a dynamic steady state, which we then simulate and analyze for 2 more generations by recording all instantaneous center-of-mass forces ${{\bf{F}}}_{i}^{{\rm{cm}}}$ (as in Eq. (3)) and all individual interaction forces between all cells every 0.01 generations. Figure 4a, d shows snapshots of the steady state for rod and disk cells, respectively, with interaction forces represented as lines connecting cell centers and attack points on cell boundaries. At any instant in time, these interaction forces—and consequently the total center-of-mass forces—have different magnitudes, despite leading to an on-average smooth outward flow of the growing aggregate. The corresponding probability distributions of the magnitudes of these forces during the observation period of 2 generations are shown in Fig. 4b, e.

These distributions indicate that, in both models, cells experience a similar range and distribution of center-of-mass forces. This is consistent with the similarity of the two model definitions in terms of cell shape, size and mobility: identical growth rates imply a similar area production rate, which in turn requires similar expansion velocities in steady state in different parts of the colony. These velocities then translate to similar average center-of-mass forces and also distributions, if the fluctuations are not larger than the spatial differences between different locations. In contrast, individual interaction forces depend on the concrete force laws employed in each model. Although they are based on Hertzian repulsion in both cases, according to Eqs. (20 and 24), two rod cells can have at most one interaction force, while two disk cells can have up to four (practically: three) interactions which are, in addition, modulated by the softness factor, Eq. (22). While the increased number should generally result in weaker overall interaction forces, Fig. 4e also shows a difference in the shape of the interaction force distribution compared to Fig. 4b, with more dominant weak force contributions and a stronger exponential-like decay towards high forces for disk cells.

The long tails in the interaction force distribution for rod cells are consistent with strong fluctuations and force peaks described before (cf. Fig. 3). However, the former could also simply be due to the different number and attack points of the interaction forces. To test more directly whether the aberrant fluctuations also manifest on a population level or are minor compared to physical fluctuations, we calculated the integrated squared difference between consecutive force distributions 0.01 generations apart, i.e., $\Delta {P}^{2}={\int }_{0}^{\infty }{[{p}_{t}(F)-{p}_{t-0.01}(F)]}^{2}\,{\rm{d}}F$, as shown in Fig. 4c, f. While the absolute magnitude of *ΔP*^2^ depends on the size of the simulation, the time interval and the resolution with which the distributions are calculated, its relative magnitude for constant numerical parameters can be taken as a measure for temporal force fluctuations. The results show that fluctuations in interaction forces are much weaker in the disk cell model compared to the rod cell model. This might not be entirely surprising given that interaction forces are distributed over a much wider range in the rod cell case, and the number of interaction forces is lower, leading to stronger fluctuations. Nevertheless, we can also observe a reduction in fluctuations of the center-of-mass forces. Since these distributions were similar for both models (as well as the number of cells and, therefore, forces) and center-of-mass forces translate directly into motion, we can indeed conclude that the smoother nature of the disk cell model translates to smoother dynamics on the collective level.

#### Collective dynamics, orientational order, angle statistics

As we keep the average growth rate constant for all particles throughout the simulation, without any form of local cell removal, the system would grow exponentially indefinitely. As this is both unrealistic and computationally unachievable, we set up simulations inspired by experimental work on microfluidic devices^17,18,61,62^, which feature the active proliferation region bounded by a mix of confining walls and absorbing outlets. In these devices, a steady stream of nutrient-rich fluid near the outlets provides for continued growth and removal of cells that leave the active region by washing them away. Consequently, we use circular simulation domains with indiscriminate removal at the perimeter based on the center-of-mass position, thus allowing indefinite isotropic expansion with finite maximal pressure in the center and also enabling time-averaged measurements in steady state. However, it should be noted that other biophysical scenarios might require different approaches, such as a modified growth law or cell removal (apoptosis) triggered stochastically or by biochemical or mechanical cues (see section “Interactions” for convenient access to the local mechanical environment).

**Fig. 5:**
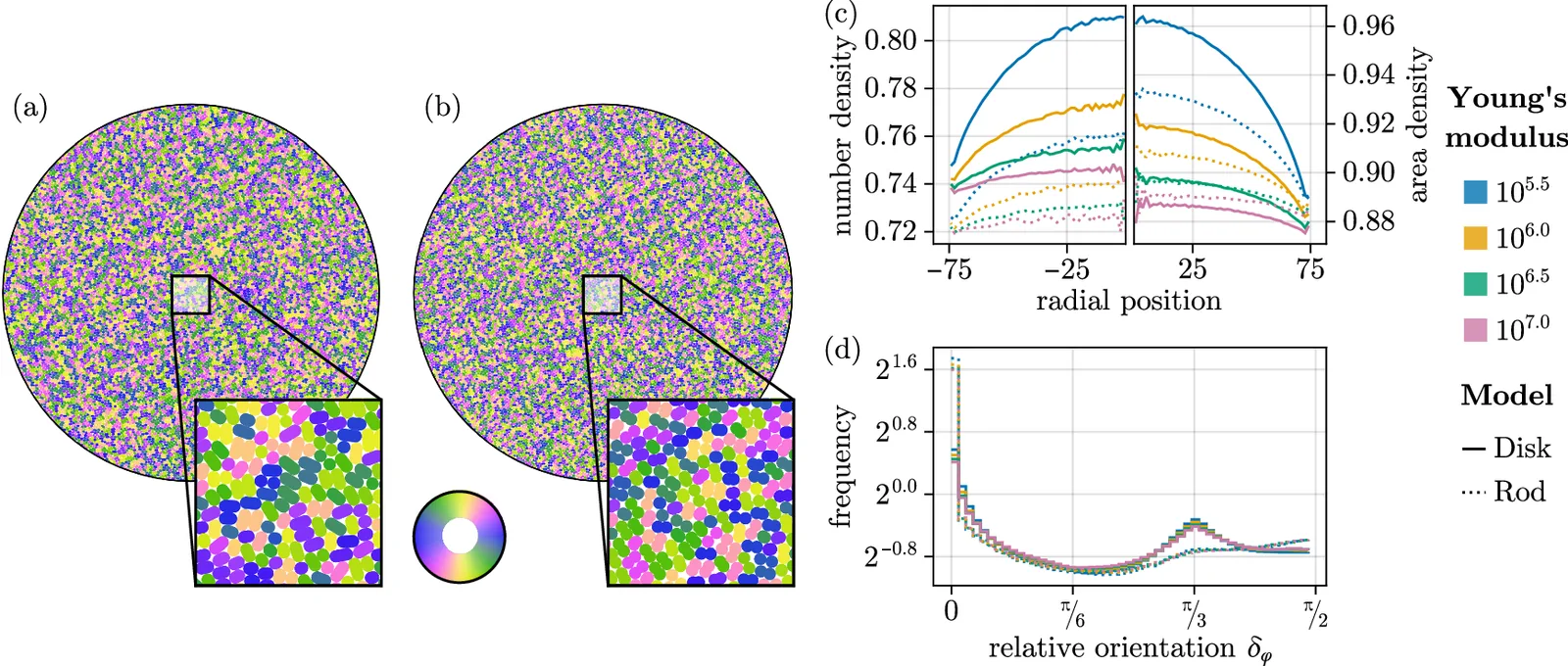
Collective dynamics in radially expanding colonies. **a** Snapshot of a circular colony of radius 75, for rod cells with parameters *R* = 0.5, *Y* = 10^7^, *η* = 0.05 and growth rates chosen uniformly from 0.75 to 1.25. For easy comparison to disk cell simulations, the division length is set to $l^{\rm{max}}=4R$. Cells are color-coded by orientation. **b** Same as (**a**) with disk cells. Parameters unchanged. **c** Number density (left) and area density (right) in the steady state as a function of radial position for disk and rod model simulations (solid and dotted lines, respectively) with different Young’s moduli, averaged over eight realizations from *t* = 15.0–30.0. All other parameters are chosen as in (**a**). **d** Frequency density of relative orientations of overlapping neighbors in the same simulations.

We initialize our simulations with four cells of random orientation and growth progress placed in the center of the system, and let the colony expand until it densely fills the entire domain (at ~12 generations). We then simulate this steady state until reaching *t* = 30 generations. For the analysis, we discard the first 15 generations to ensure that the transient expansion phase is excluded. For better statistics, we run these simulations eight times for each parameter set, with different random seeds and initial conditions for every realization. Figures 5a, b show the final state of a simulation for rod and disk cells, respectively.

The main difference between the disk and rod models is their steric interaction laws. Therefore, we first compare the steady-state densities at equal Young’s modulus *Y*. Figure 5c shows the time-averaged number density profile for disks and rods as a function of radial position, measured by counting cell centers of mass within concentric bins of width 1.5. We observe a parabolic profile, taking its maximum at the domain center and falling off towards the outer edge. As expected, peak density decreases with increasing Young’s modulus for both cells, while density at the absorbing boundary, where stresses are low, changes only a little. Number densities for the rod model are consistently lower than those for disk cells of equivalent radius.

To see how much of this is caused by simply the difference in area between disk and rod cells of equal growth progress, we additionally compute area densities by adding the individual cell areas in a bin and dividing by the bin area. We find that while for lower Young’s moduli the area density is still lower in the rod simulations, the difference between models decreases with increasing hardness. For *Y* = 10^7^, area density for the disk model is in fact slightly lower than for the rod model everywhere in the domain, while number density is still significantly higher. This implies that while the area difference does contribute to the observed number density difference, for softer cells, there must be additional effects, such as higher overlaps or more efficient packing, that affect both density measures.

To assess the influence of the different cell shapes on the microscopic structure of a dense colony, we measure the relative orientation *δ*_*φ*_ between overlapping cells in these simulations and compute a frequency histogram, which is shown in Fig. 5d. For both models, we see a distinct peak for parallel cells (*δ*_*φ*_ = 0), corresponding to local nematic alignment. This peak is much stronger for the rod model, with almost twice as many parallel pairs as for disk cells. Intuitively, this can be explained by the flat sides of the rods, which enable and stabilize parallel cell configurations.

For both models, relative orientations between 0 and around *π*/3 occur with the lowest frequency. At larger relative angles, the frequency increases again, with a pronounced secondary peak at *π*/3 for the disk model. For even higher *δ*_*φ*_, the histogram plateaus for disk cells, while rising further towards *π*/2 for the rod model. The secondary peak at *π*/3 observed in the disk data could be interpreted as the signature of local quasi-hexagonal arrangements, in which nodes of one cell can interact with the indentation between the nodes of another. In any case, these differences are manifestations of different steric interaction rules for the two models.

Aside from these local alignment effects, it is known from the literature that dense growing systems also feature nematic order at larger scales. For particles with more extreme aspect ratios, studies report the emergence of large locally aligned patches of microdomains^20,21^. Due to the fairly low anisotropy of our particles, we cannot observe these larger structures here (Fig. 5a, b).

Another effect observed in growing nematic particles is a tendency to align with shear flows. Since the expansion flow in circular colonies is shear-free if the colony is sufficiently close to incompressibility^19^, shear-induced alignment is not visible in these systems. We therefore alter our simulation setup to induce a strong uniaxial shear flow throughout the entire colony by replacing the radially symmetric absorbing boundaries with axis-parallel absorbing boundaries in one direction and confining boundaries in the other. The global nematic alignment resulting from this rectangular channel geometry has been observed and studied from different perspectives before^17,18,22,60^.

**Fig. 6:**
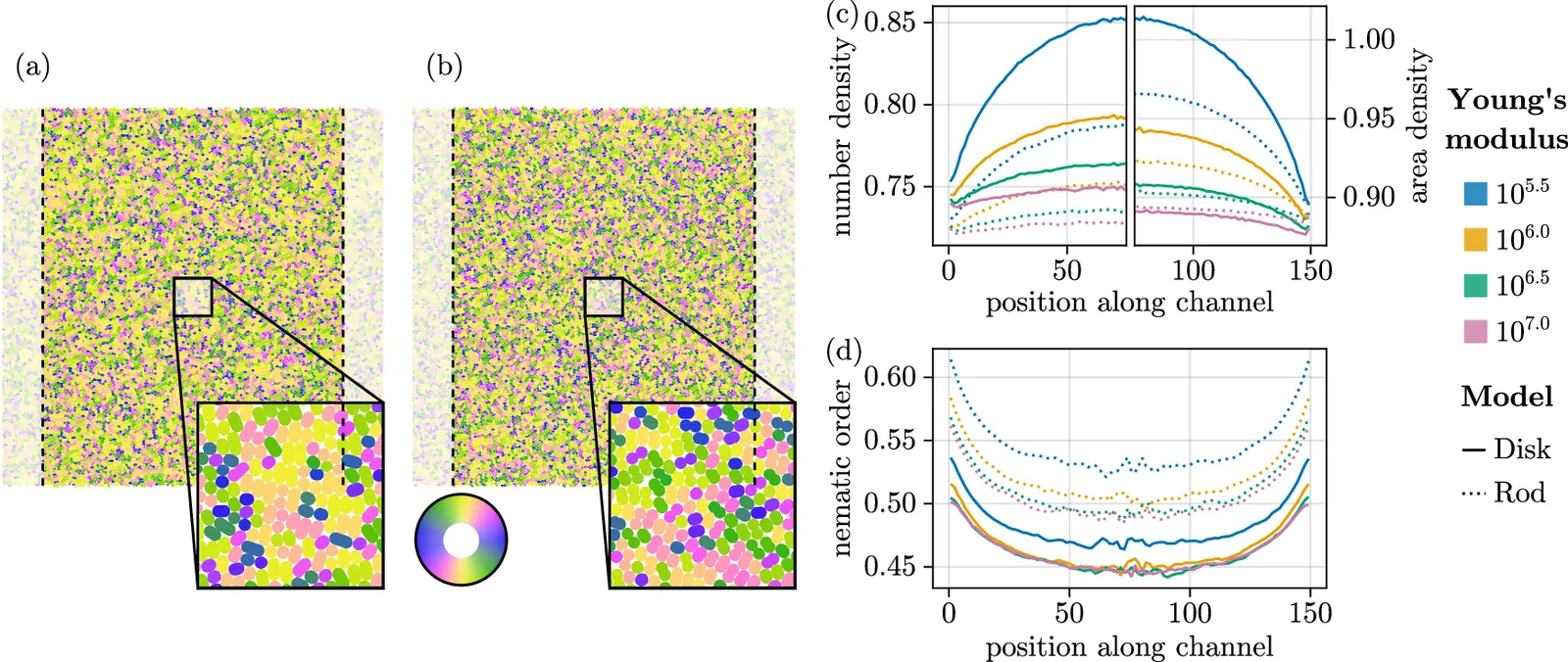
Collective dynamics in channel geometries. **a** Snapshot of an x-periodic channel of height 150 and width 120 at *t* = 30 generations, for rod cells with parameters *R* = 0.5, *Y* = 10^7^, *η* = 0.05 and growth rates chosen uniformly from 0.75 to 1.25. For easy comparison to disk cell simulations, the division length is set to $l^{\rm{max}}=4R$. Cells are color-coded by orientation. **b** Same as (**a**) with disk cells. Parameters unchanged. **c** Number density (left) and area density (right) in the steady state as a function of position along the channel axis for disk and rod model simulations (solid and dotted lines, respectively) with different Young’s moduli, averaged over eight realizations from *t* = 15.0–30.0. All other parameters are chosen as in (**a**). **d** Nematic order parameter *ξ* as a function of position along the channel axis for the same simulations.

Here, we simulate channels of size 120 × 150 units, with periodic boundaries in the x-direction and absorbing boundaries in the y-direction (Fig. 6a, b). We use the same protocol as in the previous simulations and again discard the transient phase of the colony evolution. We repeat our previous measurements of number and area density (Fig. 6c) and find that the results are qualitatively similar, although densities are higher than in the circular systems. This can be understood as a consequence of the confinement: stresses can now only be relaxed in the unconfined direction.

For both models, we can observe the expected nematic alignment with the channel direction throughout the system. This is directly visible in Figs. 6a, b where cells are color-coded by orientation. We quantify this with a nematic order parameter $\xi =| {\langle \exp (2i{\varphi }_{j})\rangle }_{j}|$, where the average is over individual cells. This order parameter is 0 for isotropically distributed *φ*_*i*_, and 1 for identical angles. We measure *ξ* along the channel axis, averaging over bins of height 1.5 that span the width of the channel. Figure 6d shows that the order parameter is ~0.45 to 0.6 for both models, corresponding to the visual impression of a distinct alignment bias with remaining disorder. For both models *ξ* is higher near the outlets and falls to a plateau in the center of the colony. *ξ* is systematically higher for the rod model than the disk model, which is consistent with the increased preference for local nematic alignment observed in the circle domain.

With increasing Young’s modulus, the order parameter profiles are shifted to lower values, while the difference between outlet and center remains similar. Interestingly, we observe that while the density profiles still change significantly even between the two highest Young’s moduli we simulate (*Y* = 10^6.5^ and 10^7^), the order parameter profiles change very little and appear to converge.

#### Connection to tissue modeling in two and three dimensions

**Fig. 7:**
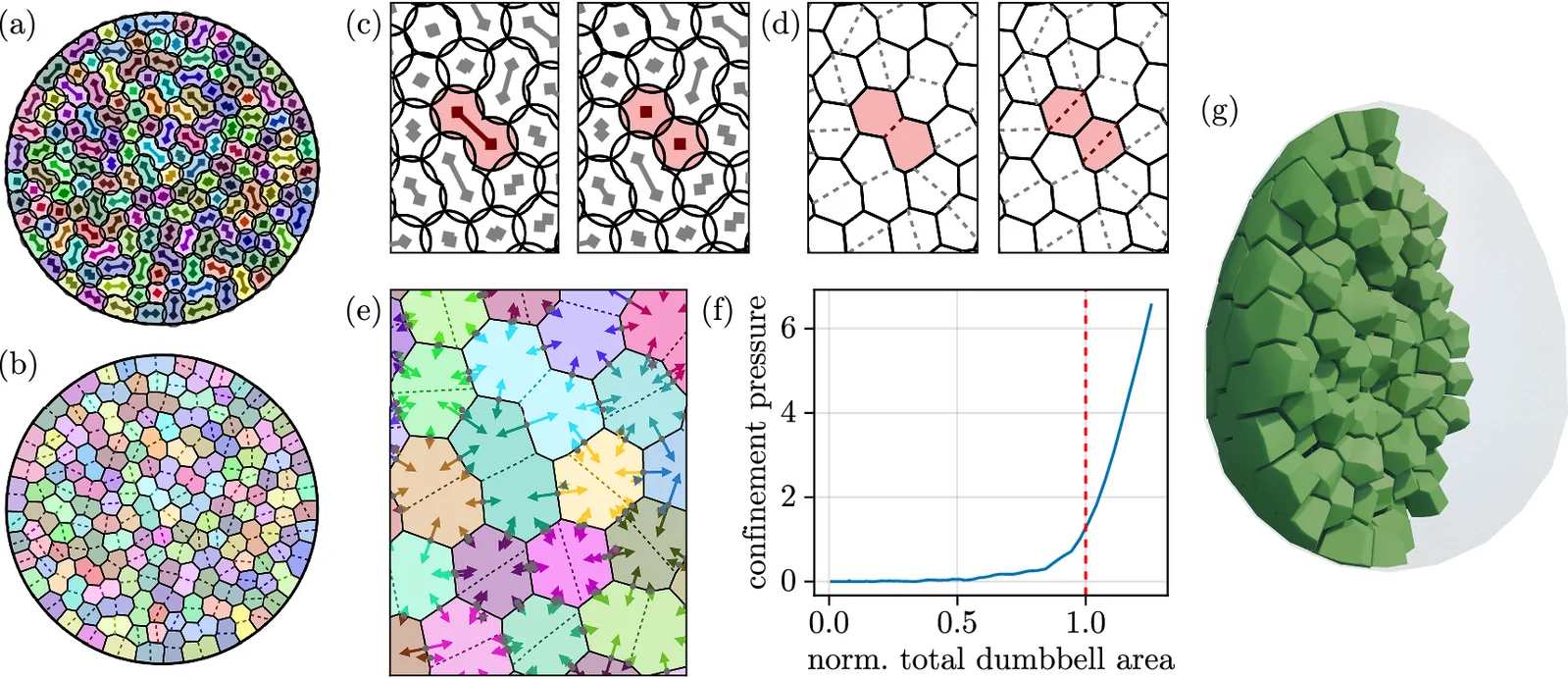
Confluent-tissue interpretation of the disk cell model in two and three dimensions. **a** Snapshot of disk cells with parameters *R* = 0.5, *Y* = 50.0, *η* = 5 × 10^−4^, growing in circular confinement of radius 7.0, at *t* = 7 generations. **b** Node-based Voronoi tessellation of the configuration in (**a**), with dashed lines indicating boundaries between nodes of the same cell. **c**, **d** Closeup before and after example of a division event, both in particle and Voronoi interpretation, showing the continuity of the configuration across divisions. **e** Interaction force dipoles between nodes of cells superimposed on the Voronoi tessellation. **f** Mechanical pressure exerted on the confining container (c.f. **a**) as a function of total cell area, which is calculated as the sum of all nominal dumbbell areas normalized by the area of the confining container. **g** Voronoi tessellation of a three-dimensional dumbbell particle simulation in egg-shaped confinement. Approximately half of the cells are removed to make the inner structure visible.

In the previous section, the modeling focused on the near incompressible regime that is appropriate for many types of bacterial colony dynamics where the individual shapes play an important role. However, in a regime with softer steric interactions, the dumbbell model can also serve as a simple model for confluent tissues. Appropriately scaling the repulsion forces and friction gives control over reorganization timescales and allows for gap-free space-filling configurations due to larger overlaps on average. Here, for these overlaps to be maintained, we let the colony of cells grow in a confining container. In the final confluent configuration, this container could represent the eggshell of a developing embryo, or, by making the boundary dynamic itself, surrounding tissue. The gap to more typical tissue models in this densely packed regime is then easily bridged by computing the Voronoi tessellation on the node centers of dumbbells. Thanks to the continuous separation dynamics in the particle model, the Voronoi cells also evolve continuously with even splits at division events. A sample snapshot of such a particle simulation is shown in Fig. 7a with the corresponding tessellation in Fig. 7b. Panels c and d show particles and Voronoi cells immediately before and after a single division event, illustrating that the surrounding configuration remains unchanged while the dividing Voronoi cell splits along the divider between nodes. The confinement allows the aggregate to develop a static pressure, similar to homeostatic pressure in tissues^3,57^, which could be used for feedback control of cellular activity and growth. In Fig. 7e, the reciprocal repulsion forces are shown as force dipoles together with their attack points, which, by construction, mostly lie on the Voronoi boundaries (except for the case of strongly overlapping disks, which may interact with their topologically next-nearest neighbors). The pressure calculated from the interaction forces with the boundary (Fig. 7f) shows a characteristic dependence on area fraction, measured as the sum of all dumbbell areas in their completely relaxed state normalized by the container area: appreciable pressure can only be measured once a critical area fraction below 1.0 is reached, indicating tissue confluency, while rapidly rising beyond this point.

One particular advantage of this approach to tissue modeling is that it easily generalizes to three dimensions, which is more involved for vertex-based models^45–47^. To demonstrate this, we present a three-dimensional extension of the dumbbell model that can generate core aspects of bulk tissue phenomena, again shown here in confinement, such as for embryonic development. Extending the dumbbell shape itself to three dimensions is straightforward: spheres replace the disks in the construction, and an orientation vector must be used rather than a simple in-plane orientation *ϕ*_*i*_. Correspondingly, the equation of motion for *ϕ*_*i*_ is replaced by an equation for the orientation vector ${\widehat{{\bf{e}}}}_{i}$

25 $${\partial }_{t}{\widehat{{\bf{e}}}}_{i}={\mu }_{{\rm{rot}}}\cdot ({\bf{T}}\times {\widehat{{\bf{e}}}}_{i})$$

Here, torque **T** is a pseudovector, as Eq. (18) now uses the three-dimensional cross product. The two-dimensional equation of motion for *φ* can be recovered by inserting **T** = (0, 0, *T*) and restricting dynamics to the *xy*-plane.

Of course, indefinite volume growth within a finite container cannot occur in practice and regulation mechanisms such as pressure or cell volume regulation would need to be introduced for studying long-time dynamics. Other additional modeling aspects, such as generation-dependent growth rate variations and adhesion mechanisms, can easily be added to investigate different aspects of these mechanobiological dynamics. However, the above already captures the most essential ingredients for our demonstration purpose. As an example, a single soft cell grows for approximately nine doubling times in an egg-shaped confinement, densely filling the system.

In the same way as in two dimensions, it is now possible to compute a bounded Voronoi tessellation in three dimensions, as shown in Fig. 7g, giving direct access to statistical analyses of effective volume distributions and cell coordination numbers.

## Discussion

In this study, we introduced a minimal model for growing and dividing cells with a focus on mechanical consistency and continuity. While the smallest mechanical unit of our model has radial symmetry (as in many other models in the literature), we explicitly combined two such units as nodes of an entire disk cell to achieve force continuity across divisions, internally between the two separating compartments, as well as with interaction partners. While this approach predictably leads to smoother behavior compared to models with instantaneous replacement of the mother cell by differently-shaped children, we showed that it also eliminates unphysical force jumps upon division present in an established model with incremental elongation and similar cell shape. The elimination of these jumps also led to reduced fluctuations in an entire cell colony, while larger simulations revealed similar collective dynamics. This should enable future studies to focus on physically meaningful forces and fluctuations, e.g., in stress calculations^17,63^, or when cell activity itself is stress-dependent^3,57^.

In particular, this is relevant for the analysis of center-of-mass trajectories of individual cells on short timescales. Large unphysical displacements compensating for model discontinuities disrupt displacement statistics such as mean-squared displacements, van Hove functions or velocity correlations. Moreover, extracting statistically fair trajectories from arbitrary observation windows requires that cell identities are preserved and compartments can be tracked across divisions, which is possible in the disk cell model presented here due to the smooth transition from intracellular nodes to entire cells.

The disk cell model also avoids another pitfall when studying collective dynamics from an active-matter perspective: for accurate analysis of the stress mechanics, one has to account for *all* forces, including active contributions. In motile active matter, a prescribed active velocity *v*_*a*_ can often be converted to an equivalent active force *F*_*a*_ = *v*_*a*_/*μ* via the translational mobility *μ*, but this is conceptually difficult with proliferating systems where activity is injected in the form of growth. Even in the models with continuous expansion discussed here, this challenge required careful design choices regarding, e.g., the internal mobility for disk cells (see section “Force decomposition”) and how to account for the forces associated with a prescribed backbone length for rod cells. However, in the new disk cell model, once these choices are made, all motion results from explicitly calculated forces, including the active elongation force of the internal spring, eliminating the need to account for it separately. This explicit tracking of forces will be particularly useful for connecting particle-based simulations to hydrodynamic continuum theories of proliferating active matter^19,22,64^, where stress tensors are fundamental quantities. In particular, it will be interesting to see how our approach to growth as relaxation of an internal spring translates to an active stress with either weak or vanishing ambient nematic order. Mathematically, this treatment of growth is known from successful descriptions of plastic processes in elastic solids, where it is natural that growth must directly interact with the elastic deformation of the material^65^. Establishing a bridge back to the fluid-like behavior of active nematic models can then be done by reintroducing viscous relaxation into the elastic solid description, yielding strain and stress as a consequence of growth. An in-depth discussion of this can be found in Ref. ^66^, and, recently, a related modeling approach used explicit strain-lets added to a fluid-based nematic model^67^. We emphasize that our disk cell model, in its presented form, is intended as an adaptable general-purpose model for multicellular systems rather than for a particular cell type.

Radially symmetric interaction potentials were chosen for simplicity, with the transient dumbbell shape during elongation as a necessary consequence, and additional modifications were only introduced to achieve mechanical consistency. However, there are biological examples, such as coccus-shaped bacteria, where growth and division closely follow the dynamics introduced here for our disk cell model^68^, as well as counterexamples such as snapping division, where discontinuities might be physical at a certain level of description^69^. As we have seen in the section “Collective dynamics, orientational order, angle statistics”, the collective dynamics in terms of orientational order and packing closely resemble those of short rod cells, independent of the exact shape. One could therefore also consider our model as a smooth drop-in replacement for modeling generic cells which exhibit anisotropy upon division. In addition, in dense regimes, the model combined with a node-based Voronoi mapping admits a confluent-tissue interpretation in two and three dimensions (see section “Connection to tissue modeling in two and three dimensions”), providing access to interaction forces and effective cell volumes as well as tissue-level observables such as pressure under confinement.

Sticking to the expressed goal of a minimal force-continuous model, we let cells grow linearly in time. A natural extension for future research is to reinterpret the two nodes of our model cell as either separating nuclei during mitosis or separating compartments during cytokinesis, since they both potentially involve the exertion of forces on the environment through various active cytoskeleton components. In this setting, plausible next steps would be to introduce variations in the cell’s life cycle dependent on the mechanical environment (as alluded to in “Growth and division” and “Interactions” within the “Disk cell model” section) or decouple volume growth from shape changes, easily possible given the modular nature of the model. Additional stochastic variations in positional, orientational or growth-progress space, as well as asymmetric division, can be added through simulated noise processes. By serving as the “mechanical backbone” for extensions with additional mechanical, chemical or regulatory features and with its complementary interpretation as a confluent tissue, we hope that our model will facilitate the characterization of growing multicellular systems in the framework of active matter and non-equilibrium statistical physics, while also bridging the gap to tissue morphogenesis and embryogenesis.

## Methods

### Numerical methods

All models are implemented according to the definitions in section “Disk cell model” and section “Rod cell model” with parameters specified in the figure captions. The simulation framework InPartS (see Code Availability) is capable of performing time integration either with a fixed time step or with an adaptive scheme controlling the step size by setting a desired maximum displacement $\Delta {x}_{\max }$ (typically 10^−3^ to 10^−1^ cell diameters depending on application). For the quantitative results in Figs. 3 and 4, we use a fixed time step of Δ*t* = 10^−5^ to exclude the possibility that force fluctuations and the dynamically chosen time steps interfere with each other and compromise the force measurements. For Figs. 5 and 6, we use $\Delta {x}_{\max }=0.005$, with the adaptive time step capped at $\Delta {t}_{\max }=0.01$, while for the high-density simulations in Fig. 7, we use $\Delta {x}_{\max }=0.0001$ and $\Delta {t}_{\max }=0.0001$.

## Data Availability

Data sharing is not applicable to this article, as only the dynamics of the mathematical models presented in the article itself were analyzed. For convenience, an implementation of the computational models to reproduce the findings of this study is provided (see Code Availability).

## Code availability

Reference implementations in the Julia programming language^70^ of both models presented here (with several additional features) are available via the package InPartSBiome.jl at http://biome.inparts.org or through the persistent identifier in ref. ^71^, together with example simulation scripts. These implementations are based on InPartS.jl, available at http://inparts.org or through the persistent identifier in Ref. ^72^, which is developed in our group and provides a common modeling framework for **In**teracting **Part**icle **S**imulations with ready-made tools for the implementation of various mechanical interaction laws, out-of-the-box import/export and visualization.
